# 2,2,6,6-Tetra­methyl­piperidinium penta­chloro­benzene­thiol­ate

**DOI:** 10.1107/S1600536808025877

**Published:** 2008-08-20

**Authors:** Katarzyna Baranowska, Natalia Piwowarska

**Affiliations:** a Department of Inorganic Chemistry, Faculty of Chemistry, Gdańsk University of Technology, 11/12 G. Narutowicz Street, 80952-PL Gdańsk, Poland

## Abstract

In the crystal structure of the title compound, C_9_H_20_N^+^·C_6_Cl_5_S^−^, two cation–anion pairs are linked by N—H⋯S hydrogen bonds to produce a cyclic aggregate of *R*_4_^2^(8) type. The dimers are interconnected *via* π–π stacking [centroid–centroid distance = 3.851(2) Å] and weak C—H⋯Cl hydrogen-bonding inter­actions.

## Related literature

For the structures of similar salts and comparison of bond distances, see: Baranowska *et al.* (2008 ▶); Dołęga *et al.* (2008 ▶); Baranowska (2007 ▶); Pladzyk & Baranowska (2007 ▶); Baranowska, Chojnacki, Konitz *et al.* (2006 ▶); Baranowska, Chojnacki, Gosiewska & Wojnowski (2006 ▶); Baranowska *et al.* (2003 ▶). For the graph-set description of hydrogen-bonding patterns, see: Bernstein *et al.* (1995 ▶); Etter (1990 ▶). For synthesis techniques, see: Perrin & Armarego (1988 ▶).
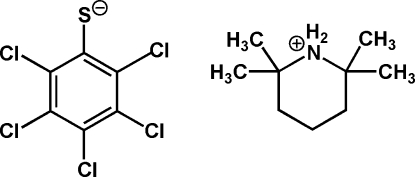


## Experimental

### 

#### Crystal data

C_9_H_20_N^+^·C_6_Cl_5_S^−^
*M* *_r_* = 423.63
Triclinic, 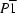

*a* = 8.4230 (5) Å
*b* = 10.5081 (4) Å
*c* = 11.6142 (6) Å
α = 110.946 (4)°
β = 102.614 (4)°
γ = 95.286 (4)°
*V* = 920.39 (8) Å^3^
*Z* = 2
Mo *K*α radiation
μ = 0.90 mm^−1^
*T* = 120 (2) K
0.21 × 0.14 × 0.09 mm

#### Data collection

Oxford Diffraction KM4 CCD diffractometer
Absorption correction: analytical (*CrysAlis RED*; Oxford Diffraction, 2006 ▶) *T*_min_ = 0.779, *T*_max_ = 0.866
5583 measured reflections
3161 independent reflections
2930 reflections with *I* > 2σ(*I*)
*R*_int_ = 0.019

#### Refinement

*R*[*F* ^2^ > 2σ(*F* ^2^)] = 0.034
*wR*(*F* ^2^) = 0.097
*S* = 1.21
3161 reflections
279 parameters
All H-atom parameters refined
Δρ_max_ = 0.67 e Å^−3^
Δρ_min_ = −0.44 e Å^−3^

### 

Data collection: *CrysAlis CCD* (Oxford Diffraction, 2006 ▶); cell refinement: *CrysAlis RED* (Oxford Diffraction, 2006 ▶); data reduction: *CrysAlis RED*; program(s) used to solve structure: *SHELXS97* (Sheldrick, 2008 ▶); program(s) used to refine structure: *SHELXL97* (Sheldrick, 2008 ▶); molecular graphics: *ORTEP-3 for Windows* (Farrugia, 1997 ▶); software used to prepare material for publication: *WinGX* (Farrugia, 1999 ▶).

## Supplementary Material

Crystal structure: contains datablocks I, global. DOI: 10.1107/S1600536808025877/im2076sup1.cif

Structure factors: contains datablocks I. DOI: 10.1107/S1600536808025877/im2076Isup2.hkl

Additional supplementary materials:  crystallographic information; 3D view; checkCIF report

## Figures and Tables

**Table 1:** Hydrogen-bond geometry (Å, °)

*D*—H⋯*A*	*D*—H	H⋯*A*	*D*⋯*A*	*D*—H⋯*A*
N1—H1*B*⋯S1^i^	0.87 (2)	2.44 (2)	3.301 (2)	170 (2)
N1—H1*A*⋯S1	0.90 (2)	2.39 (2)	3.226 (2)	157 (2)
C14—H14*C*⋯Cl1^ii^	0.91 (3)	3.02 (2)	3.803 (2)	145 (2)
C15—H15*B*⋯Cl1	0.93 (2)	2.88 (2)	3.748 (2)	156 (2)
C13—H13*B*⋯Cl3^iii^	0.98 (2)	3.02 (2)	3.905 (2)	151 (2)
C13—H13*C*⋯Cl4^iv^	0.98 (2)	3.08 (2)	3.782 (2)	129 (2)
C9—H9*B*⋯Cl4^iv^	0.95 (2)	2.92 (2)	3.708 (2)	141 (2)
C15—H15*C*⋯Cl4^v^	0.94 (2)	2.94 (2)	3.646 (2)	133 (2)
C8—H8*B*⋯Cl5^vi^	0.89 (3)	2.87 (3)	3.748 (2)	169 (2)
C10—H10*A*⋯Cl5^i^	0.97 (2)	3.02 (2)	3.966 (2)	167 (2)

Symmetry codes: (i) 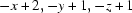; (ii) 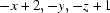; (iii) 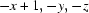; (iv) 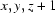; (v) 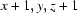; (vi) 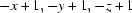.

**Fig. 1.**
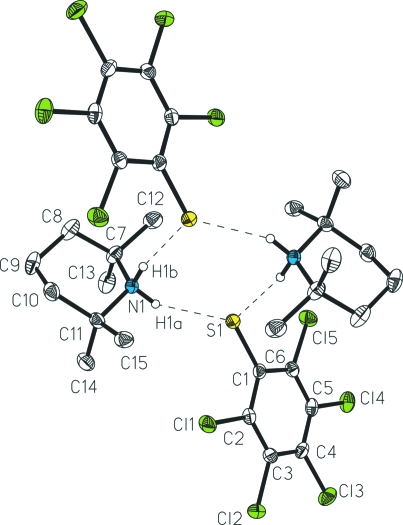
Structure of [C6Cl5S(-)H2N(+)C5H6Me4]2, showing the atomic numbering scheme. Displacement ellipsoids are drawn at the 50% probability level. C-bound H atoms have been omitted for clarity.

**Fig. 2.**
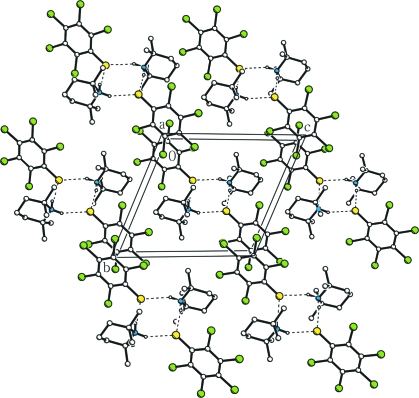
The crystal packing of (I), viewed approximately down the a axis.

## supplementary crystallographic information

### Comment

The crystal structure of the title compound shows an asymmetric unit consisting of one pentachlorobenzenethiolate anion and one 2,2,6,6-tetramethylpiperidinium cation. The ammonium thiolate forms a dimer [C_6_Cl_5_S^(-)^ H_2_N^(+)^C_5_H_6_Me_4_]_2 _(Fig. 1) in which four charge-assisted ^(+)^N—H···S^(-)^ hydrogen bonds form a stable core. This pattern of an eight-membered ring system with four donors and two acceptors is known as *R*_4_^2^(8), using Etter's graph set analysis (Etter, 1990; Bernstein *et al.*, 1995). In the crystal the dimers pack as seperate units bound together by van der Waals forces and weak C—H···Cl hydrogen bonds (Fig. 2). Similar (thiol-amine)_2_ ring formation has been observed in other ammonium salts (Baranowska *et al.*, 2008; Baranowska, 2007; Baranowska, Chojnacki, Konitz *et al.*, 2006; Baranowska, Chojnacki, Gosiewska & Wojnowski, 2006). The dimers are interconnected *via*π–π stacking interactions between *Cg*1 and *Cg*2, where *Cg*1 is the centroid of the C1–C6 ring and *Cg*2 is the centroid of the C1–C6 ring at (1-x, -y, -z). The centroid-to-centroid (CC) distance is 3.851 (2) Å and the angle subtended by the plane normal to CC is 25.03°. Interactions of the C—H···Cl type are weak with the shortest H···Cl distance measuring to 2.86 Å.

The N···S distances lie in the range 3.226 (2)–3.301 (2) Å and are therefore comparable with values observed in zinc and cobalt silanethiolates complexes (Dołęga *et al.*, 2008; Pladzyk & Baranowska, 2007) or aromatic thiolates (Baranowska, 2007; Baranowska *et al.* 2003).

### Experimental

All manipulations were carried out under an atmosphere of nitrogen using standard Schlenk techniques. The solvents were purified and dried by standard methods (Perrin & Armarego, 1988).

C_6_Cl_5_SH (0.570 g, 2 mmol) was dissolved in tetrahydrofurane (*ca* 10 ml). Traces of impurities were removed by filtration under an argon atmosphere. Next, a portion of 2,2,6,6-tetramethylpiperidine (0338 ml, 2 mmol) was added at room temperature. The color of the mixture changed to dark red. Slow crystallization from THF at 5° C yielded yellow crystals suitable for X-ray diffraction.

### Refinement

All H atoms were located in the difference map and refined without constraints.

### Crystal data

**Table:**

C_9_H_20_N^+^·C_6_Cl_5_S^–^	*Z* = 2
*M**_r_* = 423.63	*F*_000_ = 436
Triclinic, *P*1	*D*_x_ = 1.529 Mg m^−^^3^
Hall symbol: -P 1	Mo *K*α radiation λ = 0.71073 Å
*a* = 8.4230 (5) Å	Cell parameters from 6782 reflections
*b* = 10.5081 (4) Å	θ = 2.0–32.2º
*c* = 11.6142 (6) Å	µ = 0.90 mm^−^^1^
α = 110.946 (4)º	*T* = 120 (2) K
β = 102.614 (4)º	Prism, yellow
γ = 95.286 (4)º	0.21 × 0.14 × 0.09 mm
*V* = 920.39 (8) Å^3^	

### Data collection

**Table:**

Oxford Diffraction KM4 CCD diffractometer	3161 independent reflections
Monochromator: graphite	2930 reflections with *I* > 2σ(*I*)
Detector resolution: 8.1883 pixels mm^-1^	*R*_int_ = 0.019
*T* = 120(2) K	θ_max_ = 25.1º
0.75° wide ω scans	θ_min_ = 2.0º
Absorption correction: analytical(CrysAlis RED; Oxford Diffraction, 2006)	*h* = −10→10
*T*_min_ = 0.779, *T*_max_ = 0.866	*k* = −12→12
5583 measured reflections	*l* = −13→10

### Refinement

**Table:**

Refinement on *F*^2^	Secondary atom site location: difference Fourier map
Least-squares matrix: full	Hydrogen site location: inferred from neighbouring sites
*R*[*F*^2^ > 2σ(*F*^2^)] = 0.034	All H-atom parameters refined
*wR*(*F*^2^) = 0.097	*w* = 1/[σ^2^(*F*_o_^2^) + (0.0605*P*)^2^ + 0.2069*P*] where *P* = (*F*_o_^2^ + 2*F*_c_^2^)/3
*S* = 1.21	(Δ/σ)_max_ < 0.001
3161 reflections	Δρ_max_ = 0.67 e Å^−^^3^
279 parameters	Δρ_min_ = −0.44 e Å^−^^3^
Primary atom site location: structure-invariant direct methods	Extinction correction: none

### Special details

**Table:**

Geometry. All e.s.d.'s (except the e.s.d. in the dihedral angle between two l.s. planes) are estimated using the full covariance matrix. The cell e.s.d.'s are taken into account individually in the estimation of e.s.d.'s in distances, angles and torsion angles; correlations between e.s.d.'s in cell parameters are only used when they are defined by crystal symmetry. An approximate (isotropic) treatment of cell e.s.d.'s is used for estimating e.s.d.'s involving l.s. planes.
Refinement. Refinement of *F*^2^ against ALL reflections. The weighted *R*-factor *wR* and goodness of fit *S* are based on *F*^2^, conventional *R*-factors *R* are based on *F*, with *F* set to zero for negative *F*^2^. The threshold expression of *F*^2^ > σ(*F*^2^) is used only for calculating *R*-factors(gt) *etc*. and is not relevant to the choice of reflections for refinement. *R*-factors based on *F*^2^ are statistically about twice as large as those based on *F*, and *R*- factors based on ALL data will be even larger.

### Fractional atomic coordinates and isotropic or equivalent isotropic displacement parameters (Å^2^)

**Table:**

	*x*	*y*	*z*	*U*_iso_*/*U*_eq_	
Cl1	0.93273 (6)	0.07455 (4)	0.24695 (4)	0.02718 (15)	
Cl2	0.82093 (6)	−0.14335 (4)	−0.03115 (4)	0.02729 (15)	
Cl3	0.58390 (6)	−0.08459 (5)	−0.24491 (4)	0.03127 (16)	
Cl4	0.44756 (6)	0.19129 (5)	−0.17487 (4)	0.02934 (15)	
Cl5	0.57116 (6)	0.41671 (5)	0.09814 (4)	0.02804 (15)	
S1	0.80012 (6)	0.36293 (4)	0.32284 (4)	0.02321 (15)	
C1	0.7446 (2)	0.23869 (17)	0.16726 (15)	0.0186 (4)	
C2	0.7987 (2)	0.11030 (17)	0.13157 (16)	0.0191 (4)	
C3	0.7504 (2)	0.01128 (17)	0.00666 (17)	0.0203 (4)	
C4	0.6429 (2)	0.03617 (18)	−0.08959 (15)	0.0211 (4)	
C5	0.5855 (2)	0.16113 (18)	−0.05775 (16)	0.0207 (4)	
C6	0.6375 (2)	0.26039 (18)	0.06694 (17)	0.0197 (4)	
N1	0.89358 (18)	0.38134 (15)	0.61365 (13)	0.0176 (3)	
C7	0.7556 (2)	0.43976 (19)	0.66962 (16)	0.0238 (4)	
C8	0.8123 (3)	0.4751 (2)	0.81401 (17)	0.0300 (4)	
C9	0.8679 (2)	0.3550 (2)	0.84610 (18)	0.0309 (4)	
C10	1.0115 (2)	0.31139 (19)	0.79179 (17)	0.0246 (4)	
C11	0.9685 (2)	0.26709 (17)	0.64589 (16)	0.0208 (4)	
C12	0.7388 (3)	0.5705 (2)	0.64296 (19)	0.0299 (4)	
C13	0.5913 (2)	0.3374 (2)	0.60334 (19)	0.0309 (4)	
C14	0.8482 (3)	0.12818 (19)	0.57438 (19)	0.0281 (4)	
C15	1.1256 (2)	0.25939 (19)	0.60015 (18)	0.0252 (4)	
H14B	0.758 (3)	0.125 (2)	0.611 (2)	0.030 (5)*	
H13B	0.566 (3)	0.305 (2)	0.510 (2)	0.029 (5)*	
H15C	1.177 (2)	0.190 (2)	0.6161 (19)	0.025 (5)*	
H13A	0.511 (3)	0.388 (3)	0.627 (2)	0.039 (6)*	
H13C	0.586 (3)	0.253 (3)	0.622 (2)	0.036 (6)*	
H14A	0.802 (3)	0.112 (2)	0.486 (2)	0.026 (5)*	
H15A	1.208 (3)	0.347 (2)	0.6435 (19)	0.022 (5)*	
H15B	1.102 (3)	0.235 (2)	0.513 (2)	0.037 (6)*	
H14C	0.908 (3)	0.061 (2)	0.580 (2)	0.034 (6)*	
H12B	0.838 (3)	0.636 (3)	0.679 (3)	0.049 (7)*	
H10B	1.046 (3)	0.235 (2)	0.808 (2)	0.031 (5)*	
H10A	1.105 (3)	0.388 (2)	0.8295 (19)	0.026 (5)*	
H12C	0.703 (3)	0.549 (2)	0.552 (2)	0.029 (5)*	
H9B	0.780 (3)	0.277 (2)	0.810 (2)	0.035 (6)*	
H1B	0.974 (3)	0.452 (2)	0.640 (2)	0.024 (5)*	
H12A	0.661 (3)	0.616 (2)	0.682 (2)	0.032 (6)*	
H1A	0.860 (3)	0.349 (2)	0.528 (2)	0.024 (5)*	
H9A	0.896 (3)	0.382 (2)	0.935 (2)	0.032 (5)*	
H8A	0.906 (3)	0.553 (2)	0.851 (2)	0.025 (5)*	
H8B	0.728 (3)	0.501 (2)	0.846 (2)	0.040 (6)*	

### Atomic displacement parameters (Å^2^)

**Table:**

	*U*^11^	*U*^22^	*U*^33^	*U*^12^	*U*^13^	*U*^23^
Cl1	0.0345 (3)	0.0226 (2)	0.0221 (2)	0.00649 (19)	−0.00126 (19)	0.01083 (18)
Cl2	0.0355 (3)	0.0198 (2)	0.0260 (3)	0.00788 (19)	0.0098 (2)	0.0066 (2)
Cl3	0.0350 (3)	0.0329 (3)	0.0166 (2)	0.0010 (2)	0.00332 (19)	0.00236 (19)
Cl4	0.0234 (3)	0.0430 (3)	0.0236 (2)	0.0058 (2)	0.00059 (19)	0.0187 (2)
Cl5	0.0284 (3)	0.0250 (3)	0.0323 (3)	0.01072 (19)	0.0055 (2)	0.0130 (2)
S1	0.0333 (3)	0.0174 (2)	0.0158 (2)	0.00052 (18)	0.00399 (19)	0.00531 (18)
C1	0.0201 (8)	0.0188 (8)	0.0169 (8)	−0.0004 (6)	0.0056 (7)	0.0075 (7)
C2	0.0193 (8)	0.0206 (8)	0.0187 (8)	0.0019 (7)	0.0038 (7)	0.0105 (7)
C3	0.0212 (9)	0.0178 (8)	0.0223 (9)	0.0012 (7)	0.0072 (7)	0.0081 (7)
C4	0.0215 (9)	0.0241 (9)	0.0144 (8)	−0.0020 (7)	0.0050 (7)	0.0052 (7)
C5	0.0156 (8)	0.0294 (9)	0.0195 (8)	0.0016 (7)	0.0032 (7)	0.0139 (7)
C6	0.0184 (8)	0.0200 (8)	0.0227 (8)	0.0023 (6)	0.0066 (7)	0.0104 (7)
N1	0.0209 (8)	0.0174 (7)	0.0148 (7)	0.0040 (6)	0.0035 (6)	0.0072 (6)
C7	0.0224 (9)	0.0317 (9)	0.0188 (8)	0.0106 (7)	0.0067 (7)	0.0094 (7)
C8	0.0248 (10)	0.0456 (12)	0.0189 (9)	0.0122 (9)	0.0069 (8)	0.0097 (8)
C9	0.0293 (10)	0.0452 (12)	0.0177 (9)	0.0010 (9)	0.0032 (8)	0.0150 (8)
C10	0.0260 (10)	0.0246 (9)	0.0227 (9)	0.0028 (8)	0.0004 (7)	0.0126 (7)
C11	0.0239 (9)	0.0182 (8)	0.0209 (8)	0.0057 (7)	0.0025 (7)	0.0100 (7)
C12	0.0333 (11)	0.0302 (10)	0.0270 (10)	0.0163 (9)	0.0078 (9)	0.0098 (8)
C13	0.0206 (10)	0.0465 (12)	0.0268 (10)	0.0061 (9)	0.0032 (8)	0.0175 (9)
C14	0.0321 (11)	0.0211 (9)	0.0284 (10)	0.0008 (8)	0.0012 (8)	0.0117 (8)
C15	0.0267 (10)	0.0222 (9)	0.0243 (10)	0.0086 (8)	0.0036 (8)	0.0073 (8)

### Geometric parameters (Å, °)

**Table:**

Cl1—C2	1.7286 (16)	C8—H8B	0.89 (3)
Cl2—C3	1.7228 (18)	C9—C10	1.516 (3)
Cl3—C4	1.7225 (16)	C9—H9B	0.95 (2)
Cl4—C5	1.7283 (16)	C9—H9A	0.94 (2)
Cl5—C6	1.7246 (18)	C10—C11	1.535 (2)
S1—C1	1.7377 (16)	C10—H10B	0.94 (2)
C1—C6	1.411 (2)	C10—H10A	0.97 (2)
C1—C2	1.413 (2)	C11—C15	1.528 (3)
C2—C3	1.392 (2)	C11—C14	1.530 (2)
C3—C4	1.396 (3)	C12—H12B	0.94 (3)
C4—C5	1.394 (3)	C12—H12C	0.96 (2)
C5—C6	1.391 (2)	C12—H12A	0.95 (3)
N1—C7	1.525 (2)	C13—H13B	0.98 (2)
N1—C11	1.529 (2)	C13—H13A	0.92 (3)
N1—H1B	0.87 (2)	C13—H13C	0.98 (2)
N1—H1A	0.90 (2)	C14—H14B	0.95 (2)
C7—C12	1.524 (3)	C14—H14A	0.96 (2)
C7—C13	1.528 (3)	C14—H14C	0.91 (3)
C7—C8	1.533 (2)	C15—H15C	0.94 (2)
C8—C9	1.524 (3)	C15—H15A	0.99 (2)
C8—H8A	0.98 (2)	C15—H15B	0.93 (2)
			
C6—C1—C2	115.27 (15)	C10—C9—H9A	111.4 (14)
C6—C1—S1	120.91 (13)	C8—C9—H9A	108.9 (13)
C2—C1—S1	123.81 (13)	H9B—C9—H9A	108.0 (19)
C3—C2—C1	122.83 (15)	C9—C10—C11	112.65 (15)
C3—C2—Cl1	118.24 (13)	C9—C10—H10B	112.4 (14)
C1—C2—Cl1	118.93 (13)	C11—C10—H10B	105.7 (13)
C2—C3—C4	120.12 (16)	C9—C10—H10A	109.9 (12)
C2—C3—Cl2	120.71 (13)	C11—C10—H10A	107.8 (12)
C4—C3—Cl2	119.16 (14)	H10B—C10—H10A	108.1 (18)
C5—C4—C3	118.69 (16)	C15—C11—N1	105.70 (13)
C5—C4—Cl3	120.68 (13)	C15—C11—C14	109.75 (15)
C3—C4—Cl3	120.62 (14)	N1—C11—C14	110.28 (14)
C6—C5—C4	120.59 (16)	C15—C11—C10	110.55 (14)
C6—C5—Cl4	120.17 (14)	N1—C11—C10	107.83 (13)
C4—C5—Cl4	119.24 (13)	C14—C11—C10	112.49 (15)
C5—C6—C1	122.46 (16)	C7—C12—H12B	112.2 (16)
C5—C6—Cl5	118.30 (13)	C7—C12—H12C	111.0 (13)
C1—C6—Cl5	119.22 (13)	H12B—C12—H12C	109 (2)
C7—N1—C11	120.66 (13)	C7—C12—H12A	110.5 (14)
C7—N1—H1B	104.8 (14)	H12B—C12—H12A	105 (2)
C11—N1—H1B	106.9 (14)	H12C—C12—H12A	108.8 (19)
C7—N1—H1A	109.8 (13)	C7—C13—H13B	111.6 (12)
C11—N1—H1A	106.0 (13)	C7—C13—H13A	105.4 (15)
H1B—N1—H1A	108.1 (18)	H13B—C13—H13A	106 (2)
C12—C7—N1	106.27 (15)	C7—C13—H13C	114.9 (13)
C12—C7—C13	108.56 (16)	H13B—C13—H13C	105.8 (18)
N1—C7—C13	110.89 (15)	H13A—C13—H13C	113 (2)
C12—C7—C8	110.81 (16)	C11—C14—H14B	111.3 (13)
N1—C7—C8	106.89 (14)	C11—C14—H14A	111.4 (12)
C13—C7—C8	113.20 (16)	H14B—C14—H14A	107.1 (18)
C9—C8—C7	113.09 (16)	C11—C14—H14C	106.6 (14)
C9—C8—H8A	108.4 (12)	H14B—C14—H14C	111 (2)
C7—C8—H8A	107.6 (12)	H14A—C14—H14C	109.9 (19)
C9—C8—H8B	110.8 (15)	C11—C15—H15C	110.0 (12)
C7—C8—H8B	107.1 (15)	C11—C15—H15A	113.3 (12)
H8A—C8—H8B	110 (2)	H15C—C15—H15A	107.1 (17)
C10—C9—C8	110.57 (16)	C11—C15—H15B	111.8 (14)
C10—C9—H9B	107.8 (14)	H15C—C15—H15B	105.8 (19)
C8—C9—H9B	110.1 (14)	H15A—C15—H15B	108.5 (18)

### Hydrogen-bond geometry (Å, °)

**Table:**

*D*—H···*A*	*D*—H	H···*A*	*D*···*A*	*D*—H···*A*
N1—H1B···S1^i^	0.87 (2)	2.44 (2)	3.301 (2)	170 (2)
N1—H1A···S1	0.90 (2)	2.39 (2)	3.226 (2)	157 (2)
C14—H14C···Cl1^ii^	0.91 (3)	3.02 (2)	3.803 (2)	145 (2)
C15—H15B···Cl1	0.93 (2)	2.88 (2)	3.748 (2)	156 (2)
C13—H13B···Cl3^iii^	0.98 (2)	3.02 (2)	3.905 (2)	151 (2)
C13—H13C···Cl4^iv^	0.98 (2)	3.08 (2)	3.782 (2)	129 (2)
C9—H9B···Cl4^iv^	0.95 (2)	2.92 (2)	3.708 (2)	141 (2)
C15—H15C···Cl4^v^	0.94 (2)	2.94 (2)	3.646 (2)	133 (2)
C8—H8B···Cl5^vi^	0.89 (3)	2.87 (3)	3.748 (2)	169 (2)
C10—H10A···Cl5^i^	0.97 (2)	3.02 (2)	3.966 (2)	167 (2)

Symmetry codes: (i) −*x*+2, −*y*+1, −*z*+1; (ii) −*x*+2, −*y*, −*z*+1; (iii) −*x*+1, −*y*, −*z*; (iv) *x*, *y*, *z*+1; (v) *x*+1, *y*, *z*+1; (vi) −*x*+1, −*y*+1, −*z*+1.
